# High Prevalence of Undocumented SARS-CoV-2 Infections in the Pediatric Population of the Tyrolean District of Schwaz

**DOI:** 10.3390/v14102294

**Published:** 2022-10-19

**Authors:** Teresa Harthaller, Wegene Borena, David Bante, Helena Schäfer, Oliver Strallhofer, Thomas Zöggeler, Eva Hochmuth, Luiza Hoch, Annika Rössler, Dorothee von Laer, Janine Kimpel, Barbara Falkensammer

**Affiliations:** 1 Institute of Virology, Medical University of Innsbruck, 6020 Innsbruck, Austria; 2 Dr. Strallhofer’s Office, 6200 Jenbach, Austria; 3 Department of Pediatrics I, Medical University of Innsbruck, 6020 Innsbruck, Austria

**Keywords:** SARS-CoV-2, seroprevalence, anti-spike antibody, anti-nucleocapsid antibody, neutralizing antibody, children, pediatric infection, unreported infection, humoral response, durable immune response

## Abstract

Complementing the adult seroprevalence data collected at the time of the rapid SARS-CoV-2 mass vaccination in the district of Schwaz in 2021, we set out to establish the seroprevalence of SARS-CoV-2 among the pediatric population of the district. A total of 369 children, mean age 9.9 (SD 3.4), participated in the study, answering a structured questionnaire on the history of SARS-CoV-2 infection, household contacts, symptoms and history of vaccination. We determined binding and neutralizing antibody levels using plasma samples provided. We estimated the overall prevalence of SARS-CoV-2 infection in the general pediatric population at the time of the study using the census data from Statistik Austria and daily reports of officially confirmed cases. Excluding study participants who reported a history of PCR-confirmed infection, the age-standardized seroprevalence of previously unknown SARS-CoV-2 infection among the general pediatric population of the district was 27% (95% CI: 26.1–27.8). Adding this to the officially documented cases, the true overall prevalence was 32.8% (95% CI: 31.9–33.6) in contrast to the officially documented 8.0% (95% CI: 7.5–8.5) by June 2021. This translated into a proportion of 75.7% (95% CI: 74.4–77.0) of cases being officially undocumented, suggesting a high extent of silent SARS-CoV-2 infections in the pediatric population and possibly silent transmission.

## 1. Introduction

The rapid spread of the coronavirus-disease-2019 (COVID-19)-causing, severe-acute-respiratory-syndrome-coronavirus-2 (SARS-CoV-2) around the globe has burdened health care systems worldwide with 613 million infected and 6.5 million fatalities attributed to the novel threat [1] since its first emergence in Wuhan, China, in December 2019.

Although the vast majority of infected individuals experience mild symptoms, clinical course can range from asymptomatic to fatal. Older age has been recognized as an important risk factor for more severe disease courses [2,3,4,5,6,7,8] and children have been found to be less affected by COVID-19 accordingly [9,10,11,12,13,14,15,16]. While different possible mechanisms have been suggested [17,18,19,20,21,22], the issue remains yet to be fully understood. Since mild or asymptomatic disease courses may remain unrecognized leading to underreporting, pediatric infections may be regarded as a potential hidden driver of the pandemic.

Months after its declaration as a pandemic by the World Health Organization (WHO) in March 2020, the original wildtype virus had evolved different variants of concern (VOC) [23] that appeared to have selective advantages over the wildtype strain. At the beginning of 2021, the variants B.1.1.7 (alpha) and B.1.351 (beta) had spread to Austria [24]. These new VOCs are characterized by increased transmissibility and the ability to evade host immune response, with especially the beta variant raising concerns about its immune evasion potential [25,26,27,28]. At the time, the Austrian district of Schwaz became the scene of the highest European surge of the beta variant [29]. In order to mitigate the spread of the variant, the district was chosen as a model region by the European Union, which provided 50,000 local adults with the opportunity for two doses of the Comirnaty vaccine by Biontech/Pfitzer in March/April of 2021.

Willeit et al. [30] established the SARS-CoV-2 prevalence in the district of Schwaz at the time of the implementation of this unique mass vaccination campaign, focusing on the adult population only. With the current study, we set out to establish the seroprevalence of SARS-CoV-2 antibodies among the pediatric population of the district to complete the picture. These additional insights provide a crucial piece of the overall infection status of the local population; contributing a context for the discussion of social measures, especially in regard to the pediatric population.

## 2. Materials and Methods

### 2.1. Study Population

A total of 369 children from the age of 2 up to their 16th birthday were enrolled in this study (ethical clearance was limited to this age group). Participants were invited to take part in the study at the event center of Jenbach on 26–27 June 2021 through public announcements in local newspapers and schools in the district of Schwaz. Additionally, patients of a local pediatrician’s office also had the opportunity to take part in the study in his office in Jenbach between 1 May and 30 August 2021. After providing informed consent, participants, together with their legal guardians, were asked to provide sociodemographic data as well as a history of SARS-CoV-2 infection in form of a short questionnaire (see supplementary material File S1). For serological analysis, 9 mL of EDTA blood was collected from each participant.

Study participants were eligible for enrollment if they met the following inclusion criteria: Participants at the time of enrollment had to be at least 2 years old and no older than 15 years. Participants had to be permanent residents of the district of Schwaz and could not have received any vaccine against SARS-CoV-2 at the time of enrollment to be eligible.

Children who did not meet the eligibility criteria or failed to provide questionnaire data and/or blood samples were excluded from the study. All children, who accepted the invitation to participate in the study and met the eligibility criteria were enrolled.

Additionally, a subcohort of 147 unvaccinated, previously infected individuals aged 18 and above were chosen from a set of 2474 adults who participated in a seroepidemiological study in March 2021 in the district of Schwaz [30] for comparison to the pediatric study population. All participants with a positive PCR or antigen test within the last 300 days prior to enrollment in the original study in March and no history of vaccination against SARS-CoV-2 were selected.

### 2.2. Laboratory Analysis

Serological analyses were performed using the Abbott CE-labeled SARS-CoV-2-IgG-II-Quant-Assay (Abbott, IL, USA) for detecting anti-spike (anti-S) antibodies using chemiluminescent microparticle immunoassay technology. Antibody results were provided in binding antibody units per milliliter (BAU/mL) and values above the cutoff of 7.1 BAU/mL were interpreted as positive as defined by the manufacturer.

Additionally, each plasma was tested for the presence of anti-nucleocapsid (anti-N) antibodies (including immunoglobulin G) with the Elecsys Anti-SARS-CoV-2 Assay performed on Roche Cobas e411 analyzer (Roche Diagnostics, IN, USA).

The detection of anti-N antibodies specifically confirms a history of SARS-CoV-2 infection whereas anti-S antibodies are generated in both convalescent and vaccinated individuals.

In children with detectable anti-S or anti-N antibodies, titers of neutralizing antibodies against the ancestral virus were quantified using a pseudovirus neutralization assay, as described previously [31]. In short, a replication-defective vesicular stomatitis virus encoding GFP and pseudotyped with Wuhan-1 SARS-CoV-2 spike protein was incubated with participants’ plasma and used to infect susceptible cells. Infected cells were counted in an immunospot reader. Using a non-linear regression method [32], 50% neutralization titers (IC50) were calculated. Titers of ≥1:16 were considered positive.

### 2.3. Data Analysis

As age in our study population (mean: 9.9 years) was slightly skewed towards the upper end compared with the general pediatric population of the district (mean: 8.4 years), we adjusted our results for age in order to come up with representative overall prevalence.

To avoid selection bias towards participants with suspected infections, we combined officially registered SARS-CoV-2 infection data with our findings of unreported infections in our study population. For this purpose, we excluded previously PCR-confirmed cases from prevalence analysis and counted seropositive cases among participants without a history of infection as unreported infection. Projecting these age-standardized findings onto the official census data of the district, we estimated the true cumulative prevalence among the pediatric population at the time of the study.

Descriptive statistics were used for characterizing questionnaire data as well as the humoral immune responses (mean (SD; standard deviation), geometric mean (SD) and median (IQR; interquartile range)). Students’ *t*-test or ANOVA was applied to characterize statistical significance when analyzing continuous variables. We used Mann–Whitney *U*-test and Kruskal–Wallis test for non-parametric methods. A *p*-value of <0.05 defined statistical significance. Statistical analysis was performed using SPSS (Version 25.0. IBM Corp., Armonk, NY, USA) and Graphpad Prism 9.3.0 (Graphpad Software Inc, La Jolla, CA, USA).

### 2.4. Ethical Clearance

The study was approved by the Ethics Committee of the Medical University of Innsbruck (EC numbers: 1161/2021 (children) and 1093/2021(adults)).

## 3. Results

### 3.1. Infection Status

As shown in Table 1, a total of 369 children ages 2–15 (median 10) were included in the analysis, of which 50.4% (*n* = 186) were male.

In 14.9% (*n* = 55) a previous PCR test confirmed infection was reported. In 92.7% (*n* = 51) of these, the diagnosis could be confirmed by both anti-S and anti-N seropositivity. In two cases, only anti-S antibodies could be detected and in two further cases (samples taken at days post-infection: 117 and 237, respectively) no SARS-CoV-2 antibodies were detected at all.

Some 85.1% (*n* = 314) reported having never been tested positive for SARS-CoV-2 by PCR before, of which 74.8% (*n* = 235) also tested negative for both anti-S and anti-N antibodies in our study. In 24.8% (*n* = 78) of children without documented history of infection; however, anti-S antibodies were detected and 67 of these children also tested positive for anti-N antibodies. One participant tested positive for anti-N antibodies only. These seropositive cases without positive PCR (*n* = 79, 25.2%) constitute the proportion of underreported cases in this study cohort (see also Supplementary Table S1).

### 3.2. Household Infections

A total of 139 (37.7%) children reported a history of household infections, of which 98 (70.5%) declared no history of PCR-confirmed infection of their own.

Among all children with no history of previous infection (*n* = 314), the proportion of seropositivity was significantly higher (*p <* 0.00001) in children with a history of household infection (51%, *n* = 50/98) as opposed to children without household contact (13.4%, *n* = 29/216).

### 3.3. Characterizing PCR-Confirmed Infections

Almost all subjects who reported a previous infection experienced asymptomatic or mild clinical course: 30.9% (*n* = 17) reported no symptoms, 16.4% (*n* = 9) reported being bedridden for at least three days and no participants reported requiring hospitalization, although 2 children (3.6%) reported difficulty breathing (Table 2). Most prevalent symptoms included fever (*n* = 20, 36.4%), cough (*n* = 17, 30.9%), dysgeusia or loss of smell or taste and sore throat (*n* = 10, 18.2% each), followed by abdominal pain/diarrhea and difficulty breathing (*n*= 2, 3.6% each). Adults reported asymptomatic infection in 9.5% (*n* = 14), being bedridden for at least three days in 44.2% (*n* = 65) of cases and non-ICU hospitalization in 2% (*n* = 3), with no participants requiring ICU-treatment.

Most infections in the pediatric study population occurred in the autumn and winter of 2020/2021, during the so-called “second infection wave”, a mean of 204.6 days (SD 53.5) prior to blood sampling. Adults reported an interval between infection and blood sampling of 98.9 days (mean, SD: 42.7) days, placing them in the second infection wave as well, as blood sampling of this cohort took place in March 2021 as opposed to the pediatric group in summer 2021. Thus, both cohorts were presumably exposed to the same SARS-CoV-2 variants, mostly wild-type and B.1.1.7.

### 3.4. Serological Data

**Figure 1:**
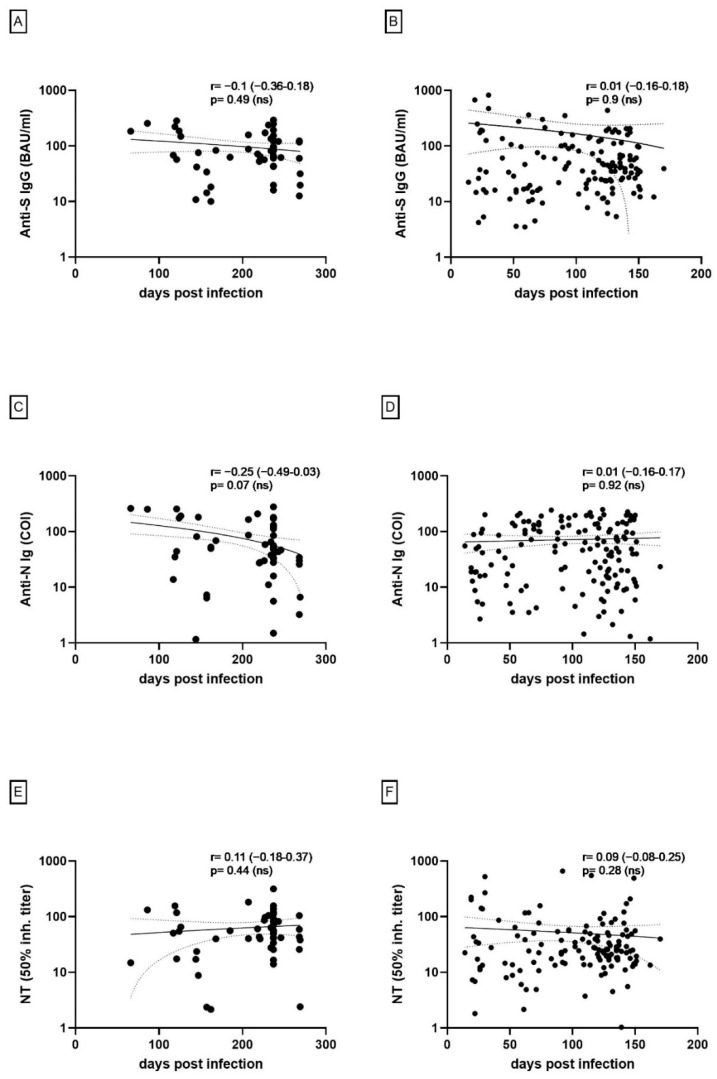
Characterizing the level of anti-SARS-CoV-2 anti-S and anti-N antibody concentration across the number of days post-infection using spearman’s correlation method: (**A**,**B**) Anti-S antibodies across time in children (**A**) and adults (**B**); (**C**,**D**) Anti-N antibodies across time in children (**C**) and adults (**D**); (**E**,**F**) Neutralizing antibody titers across time in children (**E**) and adults (**F**).

Serological analysis is summarized in Figure 1, showing relatively stable anti-S binding (Figure 1A,B) and neutralizing antibodies (Figure 1E,F) in both children and adults after a maximum of 269 and 170 days, respectively. Anti-N antibodies appeared to decline in the pediatric cohort as opposed to a steady level observed in adults (Figure 1C,D).

Differences between pediatric and adult levels of Anti-S (*p* = 0.0371) and neutralizing antibodies (*p* = 0.0002) were statistically significant, whereas differences in Anti-N antibody levels (*p* = 0.7838) did not reach statistical significance. Additionally, a significant correlation could not be established for either neutralizing antibody or binding antibody levels across the number of days post-infection in either pediatric or adult cohorts.

### 3.5. Undocumented SARS-CoV-2 Infections and Age-Standardized Seroprevalence for the Pediatric Population of Schwaz

Undocumented infection was defined as cases that tested positive for anti-N and/or anti-S antibodies without previous PCR-confirmed infection. Excluding 55 individuals with PCR-confirmed history of infection from the analysis, we found a crude seroprevalence of undocumented infections among the pediatric study population of 25.2% (95% CI: 20.5–30.4) and an age-standardized prevalence at 27.0% (95% CI: 26.1–27.8) among the general pediatric population (Table 3).

Applied to the part of the general pediatric population of the district never officially reported to have been infected with SARS-CoV-2 (11,167 out of a total of 12,133 children of the ages 2–15 in the district of Schwaz), this age-adjusted seroprevalence of 27% translated into an estimated 3011 undocumented infections (Table 3). Adding 966 children (8% of the pediatric reference population) on official record with PCR-confirmed infections until the end of June 2021, the estimated true number of infections occurring by the time of conducting this study came to 3977 cases, leading to an estimated overall SARS-CoV-2 prevalence of 32.8% (95% CI: 31.9–33.6) with a proportion of undocumented infections of 75.7% (95% CI: 74.4–77.0) at the time.

## 4. Discussion

### 4.1. Serostatus

With this study, we present a high proportion of undetected infections among the pediatric population. Our findings regarding serostatus show comparable humoral response among children and adults [33,34] and fit into the dynamic that has been described for SARS-CoV-2 humoral response, resulting in stable levels of IgG antibodies after initial rapid decline [35]. While anti-spike IgG antibodies, containing neutralizing antibodies as a subgroup, have been found to be more robust, anti-nucleocapsid antibody waning has been reported to occur more readily [36,37], which is also reflected in our data.

### 4.2. Seroprevalence

A previous study in the adult population of Schwaz found seropositivity for SARS-CoV-2 antibodies in 24%, with an estimated proportion of unreported infections of 55.8% [30]. In the pediatric population of the same district, we found age-adjusted seropositivity rates at 32.8%, higher than the adult population. Impeding direct comparison, later testing in the pediatric population is very likely to have contributed to the higher seroprevalence since the latest sampling in our study was conducted five months after the study among the adult population. This meant up to five months of additional ongoing exposure, inevitably resulting in additional infections. Although the bulk of infections occurred at the same time as in the adult population during the second infection wave, the increased exposure time could explain higher seroprevalence numbers in the pediatric cohort. Still, the incidence among adults at the time of the pediatric study was very low, possibly owing in part to the mass-vaccination few months earlier (as well as reduced overall infection dynamics concomitant with the beginning of summer), leaving the possibility of a true discrepancy irrespective of the temporal difference [38,39].

SARS-CoV-2 seroprevalence studies in general show great heterogeneity across time and population in accordance with viral exposure risk and susceptibility of the population, making comparison difficult. When looking at the proportion of pediatric seroprevalence in a given population, previous studies found conflicting evidence regarding children’s share. Several early works have reported pediatric seroprevalence to be the lowest among age groups [40,41,42,43]. Likewise, in another Tyrolean hotspot, Ischgl, Knabl et al. found a significantly lower seroprevalence in the pediatric population of 27.1% as opposed to 45% in the adult population in April 2020 [44]. Meanwhile, especially during the second wave of infection other researchers have found the highest seroprevalence in children [45] and indeed the CDC commercial lab tracker states children and adolescents in the US have the highest seroprevalence among the population [46].

### 4.3. Silent Infections among the Pediatric Population

Most frequently reported SARS-CoV-2 symptoms (fever and respiratory symptoms, see Table 2 and Table 3) are common in pediatric patients and are not specific to the disease. Thus, they ought not to be regarded as cardinal symptoms of COVID-19 in children, especially since many pediatric SARS-CoV-2 infections remain at subclinical presentation [47,48]. This fact makes identifying infected children challenging and may lead to underreporting of infections, while also increasing the risk of virus transmission.

Asymptomatic disease course has been shown to correlate with lower viral loads [49,50,51], while high viral loads have in turn been linked to increased transmissibility [51,52]. Some studies have shown children’s viral loads to be lower or similar to adults’ levels [50,51,53,54,55], contradicting a possible mechanism for children’s “silent superspreader” potential. And indeed, a lot of real-world data do not suggest a heightened bearing of children’s role in infection transmission [55,56,57,58].

Our own findings of 27% undocumented infections in the pediatric population as compared with 15% occult SARS-CoV-2 infections found by Willeit et al. in the adult population of the district of Schwaz [30], as well as the high proportion of unreported infections of 75.7% among all infections, support the notion that pediatric infections are less likely to be detected during the acute phase. Since children in our study were tested for seroprevalence later than their adult counterparts, the higher seroprevalence among the pediatric population of the district is not necessarily at odds with the perception that children’s infections reflect community infections; the role of children in infection transmission is beyond the scope of this study though.

### 4.4. Strengths and Limitations

Serological assays used in our study have very high sensitivity and specificity, and by testing positive results for neutralizing antibodies, we added significant value to the data. A further strength of the study is the availability of data on daily reports of confirmed cases that helped us estimate occult infections at the level of the general pediatric population of the district.

However, our study population is limited in size and further studies are needed to confirm these findings. At that time, persons with positive antibody results were free from mandatory SARS-CoV-2 testing for three months when entering official institutions—introducing a motivation (bias) to participate in the study [59]. An age-selection-bias effect can also be observed in this study: Only a few parents approved of their young children’s participation, and at the same time adolescents were motivated to take part in this study as they received their antibody titers free of charge. A further limitation is the missing data among children under the age of 2 (no ethical clearance) making our finding in a way incomplete.

We acknowledge that time intervals and distribution of sampling between pediatric and adult cohorts differed measurably and made a direct comparison of antibody dynamics difficult.

Lastly, SARS-CoV-2-related symptoms were self-reported by the children together with their guardians through a questionnaire, which carried the risk of underreporting of less well-publicized symptoms. Additionally, younger children might have been less able to fully describe their symptoms.

## 5. Conclusions

Our findings support the view of generally mild or asymptomatic pediatric SARS-CoV-2 infections remaining undetected at relatively higher rates than infections in adults [30]. Additionally, our study provides valuable information about the persistence of different infection-derived SARS-CoV-2 antibodies. Not only anti-S and anti-N, but crucially also neutralizing antibodies could be detected 6 months after infection and beyond. This may signify robust protection; however, the role of rapidly emerging variants, rendering present immune protection ineffective, should be kept in mind.

## Figures and Tables

**Table 1:** Baseline characteristics and serostatus of children.

	Pediatric Study Population
**Total Number of Participants**	**369**
**Age, years (range)**	**2–15**
Mean (SD)	9.9 (3.4)
Median (IQR)	10.0 (7–13)
**Sex (%)**	
Male	186 (50.4)
Female	183 (49.6)
**PCR confirmed SARS-CoV-2 infection ^§^ (% of total)**	**55 (14.9)**
Seropositive any (%)	53 (96.4)
Anti-S positive	53 (96.4)
Anti-N positive	51 (92.7)
Neutralizing antibodies	45 (81.8)
History of household infections (% of seropositive)	41 (77.4)
Seronegative	2 (3.6)
History of household infections (% of seronegative)	0 (0.0)
**No confirmed infection (% of total)**	**314 (85.1)**
Seropositive any (%)	79 (25.2)
Anti-S positive	78 (24.8)
Anti-N positive	68 (21.7)
Neutralizing antibodies	67 (21.3)
History of household infection (% of seropositive)	50 (63.3)
Seronegative	235 (74.8)
History of household infection (% of seronegative)	48 (20.4)

^§^ Self-reported. SD= standard deviation, IQR = interquartile range.

**Table 2:** Humoral response and disease characteristics in PCR confirmed ^§^ cases children vs. adults.

	Children	Adults
**Total Number**	**55**	**147**
**Age (range)**	**3–15**	**18–75**
Mean (SD)	10.5	39.8
Median (IQR)	11 (9–13)	38 (29–50)
**Sex (%)**		
Female	30 (54.5)	96 (65.3)
Male	25 (45.5)	51 (34.7)
**Days post infection (range)**	**66–269**	**14–170**
Mean (SD)	204.6 (53.5)	98.9 (42.7)
Median (IQR)	234.0 (157.0–237.0)	114.0 (62.0–135.0)
**Clinical severity (%)**		
Hospital admission	0 (0.0)	3 (2.0)
Bedridden for ≥3 days	9 (16.4)	65 (44.2) **
Asymptomatic infection	17 (30.9)	14 (9.5)
**Symptoms (%)**	**38 (69.1)**	**133 (90.5)**
Fever	20 (36.4)	n.a.
Cough	17 (30.9)	n.a.
Dysgeusia/Loss of smell or taste	10 (18.2	n.a.
Sore throat	10 (18.2)	n.a.
Abdominal pain/Diarrhea	2 (3.6)	n.a.
Difficulty breathing	2 (3.6)	n.a.
Other *	24 (43.6)	n.a.
**Anti-S antibody status ^1^**		
Mean (SD)	96.1 (75.4)	166.9 (510.7)
Geometric Mean (SD)	59.8 (75.4)	51.45 (510.7)
Median (IQR)	72.0 (42.5–134.2)	43.0 (19.5–121.3)
**Anti-N antibody status ^2^**		
Mean (SD)	72.8 (77.7))	71.6 (66.8)
Geometric Mean (SD)	29.1 (77.7)	38.0 (66.8)
Median (IQR)	43.6 (15.9–111.8)	48.6 (14.9–116.1)
**Neutralizing antibody status ^3^**		
Mean (SD)	63.1 (56.7)	51.5 (96.3)
Geometric Mean (SD)	34.3 (56.7)	15.5 (96.3)
Median (IQR)	49.6 (25.2–89.4)	23.9 (13.4–46.2)

^§^ Self-reported; * fatigue (17); headache (10); joint pain (4); eye pain (2); vomiting, runny nose, concentration difficulty, vertigo(1); ** missing 5; ^1^ *p* = 0.0371, ^2^ *p* = 0.7838, ^3^ *p* = 0.0002 (for difference in mean antibody concentration between adults and children); SD = standard deviation, IQR = interquartile range, n.a. = not available.

**Table 3:** Characterizing study population and source population without a known history of officially reported SARS-CoV-2 infection.

Age Group	Study Participants without Positive PCR (%)	Seropositive Participants without Positive PCR	Age-Specific Crude Prevalence (%, 95% CI) *	Children in Reference Population without Registered Infection (%) **	Expected Number of Unreported Cases in Reference Population ^§^	% Expected Cases of Total Reference Population without Registered Infection ^§§^
2–6	47 (15.0)	15	31.9 (22.3–47.0)	3510 (31.4)	1120	10.03% (9.5–10.6)
7–11	117 (37.3)	34	29.1 (21.0–38.2)	3965 (35.5)	1152.2	10.32% (9.8–10.9)
12–15	150 (47.8)	30	20.0 (13.9–27.3)	3692 (33.1)	738.4	6.61% (6.2–7.1)
**2–15**	**314 (100)**	**79**	**25.2 (20.5–30.3)**	**11,167 (100)**	**3011**	**26.96% (26.1–27.8)**

* Proportion of seropositive results across age strata. ** based on daily reports of SARS-CoV-2 infection in Schwaz since the beginning of the pandemic (data obtained from AGES). ^§^ calculated as: $\frac{\mathrm{age}\;\mathrm{specific}\;\mathrm{crude}\;\mathrm{prevalence}\;X\;\mathrm{number}\;\mathrm{of}\;\mathrm{people}\;\mathrm{in}\;\mathrm{the}\;\mathrm{reference}\;\mathrm{population}\;\mathrm{with}\;\mathrm{no}\;\mathrm{report}\;\mathrm{of}\;\mathrm{previous}\;\mathrm{infection}}{100}.$ ^§§^ calculated as: $\left(\frac{\mathrm{Expected}\;\mathrm{number}\;\left(n\right)\;\mathrm{of}\;\mathrm{unreported}\;\mathrm{cases}\;\mathrm{in}\;\mathrm{the}\;\mathrm{reference}\;\mathrm{population}}{11,167}\right)\times 100$. CI = confidence interval; all 95% CIs calculated based on Clopper-Pearson exact method.

## Data Availability

Data inquiries can be directed to the corresponding author.

## Supplementary Materials

The following supporting information can be downloaded at: https://www.mdpi.com/article/10.3390/v14102294/s1: File S1: Questionnaire; Table S1: Pediatric data by serostatus.

## References

[1] WHO Coronavirus (COVID-19) Dashboard|WHO Coronavirus (COVID-19) Dashboard with Vaccination Data. https://covid19.who.int/.

[2] Zhang H., Wu Y., He Y., Liu X., Liu M., Tang Y., Li X., Yang G., Liang G., Xu S. (2022). Age-Related Risk Factors and Complications of Patients With COVID-19: A Population-Based Retrospective Study. Front. Med..

[3] Zhou F., Yu T., Du R., Fan G., Liu Y., Liu Z., Xiang J., Wang Y., Song B., Gu X. (2020). Clinical course and risk factors for mortality of adult inpatients with COVID-19 in Wuhan, China: A retrospective cohort study. Lancet.

[4] Cordero-Franco H.F., De La Garza-Salinas L.H., Gomez-Garcia S., Moreno-Cuevas J.E., Vargas-Villarreal J., González-Salazar F. (2021). Risk Factors for SARS-CoV-2 Infection, Pneumonia, Intubation, and Death in Northeast Mexico. Front. Public Health.

[5] Herrera-Esposito D., Campos G.D.L. (2022). Age-specific rate of severe and critical SARS-CoV-2 infections estimated with multi-country seroprevalence studies. BMC Infect. Dis..

[6] Poletti P., Tirani M., Cereda D., Trentini F., Guzzetta G., Marziano V., Buoro S., Riboli S., Crottogini L., Piccarreta R. (2020). Age-specific SARS-CoV-2 infection fatality ratio and associated risk factors, Italy, February to April 2020. Eurosurveillance.

[7] Wang D., Hu B., Hu C., Zhu F., Liu X., Zhang J., Wang B., Xiang H., Cheng Z., Xiong Y. (2020). Clinical Characteristics of 138 Hospitalized Patients with 2019 Novel Coronavirus—Infected Pneumonia in Wuhan, China. JAMA.

[8] Williamson E.J., Walker A.J., Bhaskaran K., Bacon S., Bates C., Morton C.E., Curtis H.J., Mehrkar A., Evans D., Inglesby P. (2020). Factors associated with COVID-19-related death using OpenSAFELY. Nature.

[9] Jiehao C., Jin X., Daojiong L., Zhi Y., Lei X., Zhenghai Q., Yuehua Z., Hua Z., Ran J., Pengcheng L. (2020). A Case Series of Children With 2019 Novel Coronavirus Infection: Clinical and Epidemiological Features. Clin. Infect. Dis..

[10] Maltezou H.C., Magaziotou I., Dedoukou X., Eleftheriou E., Raftopoulos V., Michos A., Lourida A., Panopoulou M., Stamoulis K., Papaevangelou V. (2020). Children and Adolescents with SARS-CoV-2 Infection. Pediatr. Infect. Dis. J..

[11] Dong Y., Mo X., Hu Y., Qi X., Jiang F., Jiang Z., Tong S. (2020). Epidemiology of COVID-19 among Children in China. Pediatrics.

[12] Lu X., Zhang L., Du H., Zhang J., Li Y.Y., Qu J., Zhang W., Wang Y., Bao S., Li Y. (2020). SARS-CoV-2 Infection in Children. N. Engl. J. Med..

[13] Ludvigsson J.F. (2020). Systematic review of COVID-19 in children shows milder cases and a better prognosis than adults. Acta Paediatr..

[14] Mahmood M.M., Jafarli I., Al-Barazanchi A.F., Mosa N.M., Al-Ameen Z.G.Y., Alkhanchi T. (2021). What you need to know about children′s COVID-19: A systematic review. Chin. J. Contemp. Pediatr..

[15] Mehta N.S., Mytton O.T., Mullins E.W.S., Fowler T.A., Falconer C.L., Murphy O.B., Langenberg C., Jayatunga W.J.P., Eddy D.H., Nguyen-Van-Tam J.S. (2020). SARS-CoV-2 (COVID-19): What do we know about children? A systematic review. Clin. Infect. Dis..

[16] Zimmermann P., Curtis N. (2020). Coronavirus Infections in Children Including COVID-19. An Overview of the Epidemiology, Clinical Features, Diagnosis, Treatment and Prevention Options in Children. Pediatr. Infect. Dis. J..

[17] Chou J., Thomas P.G., Randolph A.G. (2022). Immunology of SARS-CoV-2 infection in children. Nat. Immunol..

[18] Loske J., Röhmel J., Lukassen S., Stricker S., Magalhães V.G., Liebig J., Chua R.L., Thürmann L., Messingschlager M., Seegebarth A. (2021). Pre-activated antiviral innate immunity in the upper airways controls early SARS-CoV-2 infection in children. Nat. Biotechnol..

[19] Pierce C.A., Sy S., Galen B., Goldstein D.Y., Orner E., Keller M.J., Herold K.C., Herold B.C. (2021). Natural mucosal barriers and COVID-19 in children. JCI Insight.

[20] Muus C., Luecken M.D., Eraslan G., Sikkema L., Waghray A., Heimberg G., Kobayashi Y., Vaishnav E.D., Subramanian A., Smillie C. (2021). Single-cell meta-analysis of SARS-CoV-2 entry genes across tissues and demographics. Nat. Med..

[21] Schuler B.A., Habermann A.C., PLosa E.J., Taylor C.J., Jetter C., Negretti N.M., Kapp M.E., Benjamin J.T., Gulleman P., Nichols D.S. (2021). Age-determined expression of priming protease TMPRSS2 and localization of SARS-CoV-2 in lung epithelium. J. Clin. Investig..

[22] Bunyavanich S., Do A., Vicencio A. (2020). Nasal Gene Expression of Angiotensin-Converting Enzyme 2 in Children and Adults. JAMA.

[23] https://www.who.int/activities/tracking-SARS-CoV-2-variants.

[24] https://www.ages.at/mensch/krankheit/krankheitserreger-von-a-bis-z/coronavirus#c12422.

[25] Tegally H., Wilkinson E., Giovanetti M., Iranzadeh A., Fonseca V., Giandhari J., Doolabh D., Pillay S., San E.J., Msomi N. (2021). Detection of a SARS-CoV-2 variant of concern in South Africa. Nature.

[26] Walker A.S., Vihta K.-D., Gethings O., Pritchard E., Jones J., House T., Bell I., Bell J.I., Newton J.N., Farrar J. (2021). Tracking the Emergence of SARS-CoV-2 Alpha Variant in the United Kingdom. N. Engl. J. Med..

[27] Lazarevic I., Pravica V., Miljanovic D., Cupic M. (2021). Immune Evasion of SARS-CoV-2 Emerging Variants: What Have We Learnt So Far?. Viruses.

[28] Liu G., Gack M.U. (2022). SARS-CoV-2 learned the ‘Alpha’bet of immune evasion. Nat. Immunol..

[29] https://www.ages.at/en/topics/pathogenic-organism/coronavirus/SARS-CoV-2-varianten-inoesterreich/”onavirus/SARS-CoV-2-varianten-in-oesterreich.

[30] Willeit P., Kimpel J., Winner H., Harthaller T., Schäfer H., Bante D., Falkensammer B., Rössler A., Riepler L., Ower C. (2022). Seroprevalence of SARS-CoV-2 infection in the Tyrolean district of Schwaz at the time of the rapid mass vaccination in March 2021 following B.1.351-variant outbreak. Front. Public Health.

[31] Riepler L., Rössler A., Falch A., Volland A., Borena W., Von Laer D., Kimpel J. (2020). Comparison of Four SARS-CoV-2 Neutralization Assays. Vaccines.

[32] Ferrara F., Temperton N. (2018). Pseudotype Neutralization Assays: From Laboratory Bench to Data Analysis. Methods Protoc..

[33] Bonfante F., Costenaro P., Cantarutti A., Di Chiara C., Bortolami A., Petrara M.R., Carmona F., Pagliari M., Cosma C., Cozzani S. (2021). Mild SARS-CoV-2 Infections and Neutralizing Antibody Titers. Pediatrics.

[34] Garrido C., Hurst J.H., Lorang C.G., Aquino J.N., Rodriguez J., Pfeiffer T.S., Fouda G.G. (2021). Asymptomatic or mild symptomatic SARS-CoV-2 infection elicits durable neutralizing antibody responses in children and adolescents. JCI Insight.

[35] Zuiani A., Wesemann D.R. (2021). Antibody Dynamics and Durability in Coronavirus Disease-19. Clin. Lab. Med..

[36] Gallais F., Gantner P., Bruel T., Velay A., Planas D., Wendling M.-J., Bayer S., Solis M., Laugel E., Reix N. (2021). Evolution of antibody responses up to 13 months after SARS-CoV-2 infection and risk of reinfection. eBioMedicine.

[37] Siller A., Seekircher L., Wachter G.A., Astl M., Tschiderer L., Pfeifer B., Gaber M., Schennach H., Willeit P. (2022). Seroprevalence, Waning and Correlates of Anti-SARS-CoV-2 IgG Antibodies in Tyrol, Austria: Large-Scale Study of 35,193 Blood Donors Conducted between June 2020 and September 2021. Viruses.

[38] Paetzold J., Kimpel J., Bates K., Hummer M., Krammer F., von Laer D., Winner H. (2022). Impacts of rapid mass vaccination against SARS-CoV2 in an early variant of concern hotspot. Nat. Commun..

[39] Bánki Z., Seekircher L., Falkensammer B., Bante D., Schäfer H., Harthaller T., Kimpel J., Willeit P., von Laer D., Borena W. (2022). Six-Month Follow-Up of Immune Responses after a Rapid Mass Vaccination against SARS-CoV-2 with BNT162b2 in the District of Schwaz/Austria. Viruses.

[40] Vos E.R.A., Hartog G.D., Schepp R.M., Kaaijk P., van Vliet J., Helm K., Smits G., Wijmenga-Monsuur A., Verberk J.D.M., van Boven M. (2020). Nationwide seroprevalence of SARS-CoV-2 and identification of risk factors in the general population of the Netherlands during the first epidemic wave. J. Epidemiol. Community Health.

[41] Pollán M., Pérez-Gómez B., Pastor-Barriuso R., Oteo J., Hernán M.A., Perez-Olmeda M., Sanmartín J.L., Fernández-García A., Cruz I., de Larrea N.F. (2020). Prevalence of SARS-CoV-2 in Spain (ENE-COVID): A nationwide, population-based seroepidemiological study. Lancet.

[42] Stringhini S., Wisniak A., Piumatti G., Azman A.S., Lauer S.A., Baysson H., De Ridder D., Petrovic D., Schrempft S., Marcus K. (2020). Seroprevalence of anti-SARS-CoV-2 IgG antibodies in Geneva, Switzerland (SEROCoV-POP): A population-based study. Lancet.

[43] Haq M., Rehman A., Ahmad J., Zafar U., Ahmed S., Khan M.A., Naveed A., Rajab H., Muhammad F., Naushad W. (2021). SARS-CoV-2: Big seroprevalence data from Pakistan—Is herd immunity at hand?. Infection.

[44] Knabl L., Mitra T., Kimpel J., Rössler A., Volland A., Walser A., Ulmer H., Pipperger L., Binder S.C., Riepler L. (2021). High SARS-CoV-2 seroprevalence in children and adults in the Austrian ski resort of Ischgl. Commun. Med..

[45] Charlton C.L., Nguyen L.T., Bailey A., Fenton J., Plitt S.S., Marohn C., Lau C., Hinshaw D., Lutsiak C., Simmonds K. (2021). Pre-Vaccine Positivity of SARS-CoV-2 Antibodies in Alberta, Canada during the First Two Waves of the COVID-19 Pandemic. Microbiol. Spectr..

[46] CDC COVID Data Tracker: Nationwide Commercial Lab Seroprevalence. https://covid.cdc.gov/covid-data-tracker/#national-lab.

[47] de Souza T.H., Nadal J.A., Nogueira R.J.N., Pereira R.M., Brandão M.B. (2020). Clinical manifestations of children with COVID-19: A systematic review. Pediatr. Pulmonol..

[48] King J.A., Whitten T.A., Bakal J.A., McAlister F.A. (2020). Symptoms associated with a positive result for a swab for SARS-CoV-2 infection among children in Alberta. Can. Med. Assoc. J..

[49] Hall S.M., Landaverde L., Gill C.J., Yee G.M., Sullivan M., Doucette-Stamm L., Landsberg H., Platt J.T., White L., Hamer D.H. (2022). Comparison of anterior nares CT values in asymptomatic and symptomatic individuals diagnosed with SARS-CoV-2 in a university screening program. PLoS ONE.

[50] Chung E., Chow E.J., Wilcox N.C., Burstein R., Brandstetter E., Han P.D., Fay K., Pfau B., Adler A., Lacombe K. (2021). Comparison of Symptoms and RNA Levels in Children and Adults with SARS-CoV-2 Infection in the Community Setting. JAMA Pediatr..

[51] Kawasuji H., Takegoshi Y., Kaneda M., Ueno A., Miyajima Y., Kawago K., Fukui Y., Yoshida Y., Kimura M., Yamada H. (2020). Transmissibility of COVID-19 depends on the viral load around onset in adult and symptomatic patients. PLoS ONE.

[52] Bhavnani D., James E.R., Johnson K.E., Beaudenon-Huibregtse S., Chang P., Rathouz P.J., Weldon M., Matouschek A., Young A.E. (2022). SARS-CoV-2 viral load is associated with risk of transmission to household and community contacts. BMC Infect. Dis..

[53] Euser S., Aronson S., Manders I., van Lelyveld S., Herpers B., Sinnige J., Kalpoe J., van Gemeren C., Snijders D., Jansen R. (2021). SARS-CoV-2 viral-load distribution reveals that viral loads increase with age: A retrospective cross-sectional cohort study. Int. J. Epidemiol..

[54] Madera S., Crawford E., Langelier C., Tran N.K., Thornborrow E., Miller S., DeRisi J.L. (2021). Nasopharyngeal SARS-CoV-2 viral loads in young children do not differ significantly from those in older children and adults. Sci. Rep..

[55] Jones T.C., Biele G., Mühlemann B., Veith T., Schneider J., Beheim-Schwarzbach J., Bleicker T., Tesch J., Schmidt M.L., Sander L.E. (2021). Estimating infectiousness throughout SARS-CoV-2 infection course. Science.

[56] Sorg A.-L., Bergfeld L., Jank M., Corman V., Semmler I., Goertz A., Beyerlein A., Verjans E., Wagner N., Von Bernuth H. (2022). Cross-sectional seroprevalence surveys of SARS-CoV-2 antibodies in children in Germany, June 2020 to May 2021. Nat. Commun..

[57] Viner R.M., Mytton O.T., Bonell C., Melendez-Torres G.J., Ward J., Hudson L., Waddington C., Thomas J., Russell S., van der Klis F. (2021). Susceptibility to SARS-CoV-2 Infection Among Children and Adolescents Compared With Adults:: A systematic review and meta-analysis. JAMA Pediatr..

[58] Caini S., Martinoli C., La Vecchia C., Raimondi S., Bellerba F., D’Ecclesiis O., Sasso C., Basso A., Cammarata G., Gandini S. (2022). SARS-CoV-2 Circulation in the School Setting: A Systematic Review and Meta-Analysis. Int. J. Environ. Res. Public Health.

[59] https://www.aektirol.at/coronavirus/rundschreiben/rundschreiben-detail/01042021-update-aktuelle-informationen-betreffend-coronavirus-sars-cov-2.

