# Supplementary material for: High Prevalence of Undocumented SARS-CoV-2 Infections in the Pediatric Population of the Tyrolean District of Schwaz
Source: Viruses. 2022 Oct 19;14(10):2294. doi: 10.3390/v14102294 (PMC9609860; doi:10.3390/v14102294)
Supplement: Supplementary file 1 [file viruses-14-02294-s001.zip › viruses-1971854-supplementary.pdf]

Supplementary Materials "High Prevalence of Undocumented SARS-CoV-2 Infections in the Pediatric Population of the Tyrolean District of Schwaz"

Supplementary File S1. Questionnaire (*English translation)*

Questionnaire

Characterization of SARS-CoV-2 antibody response among individuals between the 2nd and 16th year of life in the district of Schwaz

Have you been diagnosed with a SARS-CoV-2 infection?

Yes No

If yes, when____

If yes: how was the infection confirmed: by PCR by antibody testing

Were you diagnosed with a variant of concern (VOC)?

Yes Which one?_______________________________

No Unknown

Did you experience any symtoms? Yes No

If yes, which: Cough Fever Difficulty breathing Sore throat Dysgeusia/ Loss of taste or smell Abdominal pain/ Diarrhea Other: ____________________

____

If yes, did your illness cause you to be bedridden for 3 or more days?

Yes No

Did you have to be admitted to hospital for a SARS-CoV-2 infection?

Yes (to ICU) Yes (non-ICU) No

Has anyone living in the same household had a SARS-CoV-2 infection?

Yes Who?_____________________________When (Date)?_______________ No

Has anyone living in the same household been vaccinated? Yes No Don´t know?

If yes: when?_______________________

With which vaccine? AstraZeneca BioNTec-Pfizer Moderna Biotech Don´t know

Would you get (your child) vaccinated if there was an approved vaccine?

Yes No Don´t know?

Supplementary Materials "High Prevalence of Undocumented SARS-CoV-2 Infections in the Pediatric Population of the Tyrolean District of Schwaz"

Supplementary Table S1. Pediatric data by serostatus.

**Total seropositives (%)**	**Seropositives n=132 (35.8)**	**Seronegatives n=237 (64.2)**
**Confirmed cases[§] (%)**	**53 (40.2)**	**2 (0.8)**
Anti-S positive	53 (100)	
Anti-N positive	51 (96.2)	
Neutralizing antibodies	45 (84.9)	
**No history of infection**	**79 (59.8)**	**235 (99.2)**
Anti-S positive	78 (98.7)	
Anti-N positive	68 (86.1)	
Neutralizing antibodies	67 (84.8)	
**Symptoms (%)**	**58 (43.9)**	**44 (18.4**)
Fever	31 (23.5)	23 (9.7)
Cough	26 (19.7)	26 (11.0)
Disgeusia/Loss of taste or smell	17 (12.9)	1 (0.4)
Sore throat	16 (12.1)	13 (5.5)
Abdominal pain/Diarrhea	5 (3.8)	3 (1.3)
Difficulty breathing	3 (2.3)	3 (234)
Other	28 (21.2)*	20 (8.4)**
Asymptomatic infection	74 (56.1)	193 (81.4)
Bedridden for ≥3 days	9 (6.8)	14 (5.9)
Hospital admission	0 (0.0)	1 (0.4)
History of household infections	91 (68.9)	48 (20.3)
**Anti-S antibody status**		
Mean (SD)	94.5 (74.7)	
Geometric Mean (SD)	0.0 (74.7)	
Median (IQR)	72.1 (42.1-131.3)	
**Anti-N antibody status**		
Mean (SD)	58.4 (63.6)	
Geometric Mean (SD)	21.9 (63.6)	
Median (IQR)	36.9 (12.8-76.6)	
**Neutralizing antibody status**		
Mean (SD)	65.4 (61.2)	
Geometric Mean (SD)	0.0 (61.2)	
Median (IQR)	47.0 (24.3-96.2)	

[§]Self reported PCR-confirmed

*Fatigue(19); Headache(12); Joint/limb pain(6); Eye pain, Nausea/Vomiting, Runny nose(2); Vertigo(1)

**Headache(10); Fatigue(6); "Flu-like", Runny nose/rhinitis(4); Vomiting, Sweats, Laryngitis(1)

SD= standard deviation, IQR= interquartile range
